# Analysis of the profiles of human endogenous retroviruses W, K, and H and the systemic inflammatory status in vitiligo patients: insights on the dynamics of endogenous retroviral expression and its interplay with inflammatory response

**DOI:** 10.3389/fcimb.2026.1739332

**Published:** 2026-04-13

**Authors:** Samuel Nascimento Santos, Denis Campos Silva, Matheus Esteves Fernandes, Michelly Damasceno da Silva, Maria Kauanne de Oliveira Santos, Carolina Nunes França, Jonatas Bussador do Amaral, Marina Tiemi Shio, Anamaria da Silva Facina, André Luis Lacerda Bachi, Luiz Henrique da Silva Nali

**Affiliations:** 1 Post-graduation Program in Health Sciences, Santo Amaro University, São Paulo, Brazil; 2 Dermatology Departament, Federal University of São Paulo, São Paulo, Brazil; 3 Ent Research Lab. Department of Otorhinolaryngology -Head and Neck Surgery, Federal University of Sao Paulo, São Paulo, Brazil

**Keywords:** cytokines, expression, HERVs, inflammation, vitiligo

## Abstract

**Introduction:**

Human endogenous retroviruses (HERVs) are ancient retroviruses that infected the germline cells of our ancestors millions of years ago. Due to the typical replication process, they have endogenized and are fixed in the host genome, and at present, HERVs comprise approximately 8% of the human genome. HERVs play several roles in human physiology, but they are also involved in certain diseases, particularly in autoimmune diseases. However, there is limited information regarding the interplay of HERVs on vitiligo. Therefore, the aim of this study was to determine the frequency and the expression levels of HERV-W, HERV-K, and HERV-H, as well as the circulating levels of the pro- and anti-inflammatory cytokines, in patients with vitiligo.

**Methods:**

Peripheral blood mononuclear cells and serum samples were collected from 30 vitiligo patients and 30 healthy subjects. The expression levels of HERV-W, HERV-K, and HERV-H were qualitatively and quantitatively assessed with real-time PCR using primers complementary to the HERV-W *env*, HERV-K *gag*, and HERV-H *pol* genes and with the 2^−ΔΔCt^ method. Circulating levels of anti-HERV-W antibodies were assessed using an “in-house” ELISA. Moreover, the systemic pro-inflammatory cytokines IL-6, TNF-α, IFN-γ, and IFN-λ1 and the anti-inflammatory cytokine IL-10 were quantified using commercial ELISA kits.

**Results:**

HERV-W *env* was upregulated in patients with vitiligo and expressed approximately 2.5-fold change when compared with healthy individuals (*p* = 0.005), whereas HERV-H *pol* and HERV-K *gag* were downregulated in these patients as the healthy controls expressed 3.5-fold change (*p* = 0.001) and twofold change (*p* = 0.005), respectively. Patients with vitiligo presented higher circulating levels of IFN-λ1 and IL-1b and ratios of IFN-λ1/IL-10 and IL-6/IL-10 (*p* < 0.05, *p* < 0.05, *p* < 0.001, and *p* < 0.01, respectively). HERV-W *env* expression was positively correlated with IFN-λ1 and IFN-γ. Brown people showed higher expression of HERV-W *env* than other ethnic groups (*p* < 0.05). Women with vitiligo not only presented higher expression levels of HERV-W *env* (*p* = 0.05) than men (*p* < 0.01), but these levels were also higher than those of HERV-H *pol* (*p* = 0.05). No differences were observed between the anti-HERV *env* antibody concentrations in vitiligo patients and healthy individuals (*p* = 0.07). Strikingly, patients with vitiligo whose lesions have worsened presented higher expression levels of HERV-W *env* higher than those with stable lesions p<0.001.

**Conclusions:**

Our findings suggest that HERV-W *env* may play a pivotal role in the immunopathogenesis of vitiligo and that it may be linked to the systemic pro-inflammatory status of the disease. Our findings also suggest that HERV-W *env* might be used as a biomarker of the disease and may be linked to the worsening of vitiligo lesions. Altogether, our results support the involvement of HERV elements in vitiligo and warrant further investigation into their mechanistic contribution.

## 1 Introduction

Vitiligo is a dermatological disease characterized by hypochromic or achromic macules that affect distinct areas of the skin due to the loss of melanocytes (Picardo et al., 2015). The prevalence of this disease is estimated to be approximately 0.5%–1% of the human population (Ezzedine et al., 2015). However, its prevalence may be higher, particularly in dark-skinned populations (Sheth et al., 2013). This disease may significantly impact the quality of life of individuals by affecting their self-esteem, especially due to the high levels of stigma perception (Grimes and Miller, 2018). The etiology of vitiligo is not fully understood; however, the pathogenesis of its lesions is believed to be driven by an immunopathological response. Evidence points to an autoimmune role in the disease. Interestingly, autoantibodies against the melanocytes have been detected in the majority of individuals with vitiligo (Sandoval-Cruz et al., 2011). In addition, CD8^+^ T cells appear to be key players by inducing melanocyte death (Fukuda, 2022; Chen et al., 2021). This is still highlighted by the higher proportions of CD8^+^/CD4^+^ T cells in the lesions (Le Poole et al., 2004). The IFN-γ concentration in the serum is also higher than that in healthy individuals (Tomaszewska et al., 2020). This scenario reveals a complex autoimmune response in patients with vitiligo mediated by both humoral and cellular roles in the disease, with an important pro-inflammatory status. Although significant findings on the pathogenesis of vitiligo have been described, there is still a lack of evidence of how this response may be triggered. Thus, one possible candidate that may be an interesting factor that might interplay a role in the autoimmune pathogenesis of vitiligo are the human endogenous retroviruses (HERVs).

HERVs are ancient retroviruses that have infected our ancestor’s germline cells in several events throughout the evolution. They were initially spread through horizontal transmission and retrotransposition, and later on, after endogenization, they were transmitted throughout the generations by Mendelian inheritance (Rangel et al., 2022). Today, we know that HERVs comprise 8% of the human genome (Griffiths, 2001). The HERV genes are mostly silenced, distributed within the human genome; however, some of these HERV genes may be expressed (Li et al., 2011). There are several pieces of evidence that point to the crucial roles of HERVs and human physiology (Ting et al., 1992; Mi et al., 2000; Medstrand et al., 2001; Søe et al., 2011; SenGupta et al., 2011). However, HERV expression is also associated with disruption in the immune response. HERVs are believed to play a key role in autoimmune diseases. The most evidence that connects the role of HERVs in autoimmune diseases is related to multiple sclerosis (MS) (Rangel et al., 2022), with the findings varying from higher transcriptional activity in patients with MS (Nali et al., 2022; Perron et al., 2012; do Olival et al., 2013) to the detection of proteins in the sclerotic plaque (Mameli et al., 2007; Mameli et al., 2012) and the development of a mouse model of MS exposed to the HERV-W *env* protein (Perron et al., 2013). A previous study with an animal model of vitiligo in Smyth line chickens demonstrated that the expression of the endogenous retrovirus genes is correlated with the disease onset and induction, suggesting that ERVs may contribute to the pathogenesis of vitiligo, particularly through autoimmune response (Sreekumar et al., 2000). However, there are no studies that focused on understanding the potential role of HERVs in the immunopathogenesis of vitiligo in humans. In this sense, the aim of this study was to assess the expression levels of HERVs and the circulating levels of anti-HERV-W antibodies, as well as the systemic inflammatory status, in patients with vitiligo.

## 2 Methods

### 2.1 Study population and ethical considerations

This is a transversal case–control study. The study population was composed of 30 individuals with vitiligo and 30 healthy individuals as a control group. Vitiligo patients were recruited from the dermatology outpatient care of Universidade Federal de São Paulo, while healthy individuals were from the blood bank of the same university. Importantly, for this study, we followed the Strengthening the Reporting of Observational Studies in Epidemiology (STROBE) guidelines for case–control studies (von Elm et al., 2008). From each patient, we collected sociodemographic and clinical vitiligo data. Importantly, patients with vitiligo were clinically diagnosed and classified following the criteria of the Vitiligo Global Issue Consensus (Ezzedine et al., 2012). Individuals enrolled in the control group were recruited considering the absence of records of autoimmune diseases in the family. This study was approved by the Ethical Committee of Universidade Santo Amaro and Universidade Federal de São Paulo under protocol no. 6.421.017 and no. 6.480.370, respectively. All volunteers were included in the study after signing a written consent, which was also approved by both ethical committees. This study was carried out in agreement with the Declaration of Helsinki and the ethical guidelines outlined in Resolutions 466/2012 and 510/2016 of the Brazilian National Health Council.

### 2.2 Blood sample collection and preparation

From each volunteer, blood samples were collected into EDTA tubes to obtain peripheral blood mononuclear cells (PBMCs) for molecular analysis and also in gel-barrier dry tubes to obtain serum aliquots, which were used in the cytokine concentration analysis and the anti-HERV-W antibody detection. PBMCs were obtained with the Ficoll-Hypaque protocol, and RNA was obtained through the TRIzol/chloroform protocol. Briefly, Ficoll-Hypaque was added into whole blood at a 1:1 proportion and centrifuged at 800 × *g* for 20 min; the upper solution (containing PBMCs) was collected. Subsequently, the solution was washed repeatedly with sterile phosphate-buffered saline (PBS) and centrifuged until complete removal of the residual erythrocytes. Dry cell pellet sample RNA was extracted using the following protocol: 1 ml of TRIzol was added into each of the cell pellet samples and up-down was performed until complete homogenization. Thereafter, 200 µl of chloroform was added and the samples were centrifuged at 10,000 × *g* at −4°C. The upper phase was completely removed (approximately 600 µl), and the RNA was then precipitated with 100% isopropanol and washed twice with 70% ethanol. RNA was resuspended in 40 µl nuclease-free H_2_O. Rigorous decontamination of genomic DNA was performed with the TURBO DNA-Free Kit (Ambion, Austin, TX, USA). Absence of contaminant genomic DNA was confirmed by real-time PCR (RT-PCR) with primers complementary to the *GAPDH* gene with the absence of reverse transcriptase. RNA was quantified and then stored at −80°C. Approximately 150 ng of RNA was used to perform the cDNA synthesis with the High-Capacity cDNA Reverse Transcription Kit (Applied Biosystems - Thermofisher Waltham, Massachusetts, EUA).

### 2.3 HERV detection and relative quantification analysis

HERVs were qualitatively (presence or absence of expression) and relatively quantified in patients with vitiligo. Briefly, RT-PCR was performed for HERV-W *env*, HERV-K *gag*, and HERV-H *pol* with the primers described in **Table 1**.

**Table 1:** Sets of oligos used for the real-time PCR assays to detect each human endogenous retrovirus (HERV) and the housekeeping gene *GAPDH*.

Oligonucleotide	Forward primer	Reverse primer
HERV-W *env* (Nellåker et al., 2006)	CCAATGCATCAGGTGGGTAAC	GAGGTACCACAGACAAAAAATATTCCT
HERV-H *pol* (Filipponi et al., 2013)	CACGTTTTATCCGTGGACCC	AGGCATCCCTGCAATGATTAA
HERV-K *gag* (Guo et al., 2022)	AGCAGGTCAGGTGCCTGTAACATT	TGGTGCCGTAGGATTAAGTCTCCT
*GAPDH* (de Jonge et al., 2007)	ACCCACTCCTCCACCTTTGAC	TGTTGCTGTAGCCAAATTCGTT

The RT-PCR mix included 0.1 μM of each primer, 1× of the PowerUp SYBR Green PCR Master Mix (Thermo Fisher, Waltham, MA, USA), and 3 μl of the normalized cDNA preparation and nuclease-free H_2_O in a final mix volume of 25 μl. For all HERVs, the cycling conditions were as follows: 95°C for 10 min, followed by 40 cycles of 95°C for 1 min, 50°C for 45 s, and 60°C for 1 min. For the GAPDH assay, the cycling conditions were: 37°C for 30 min for the cDNA construct step, 95°C for 10 min, followed by 40 cycles of 95°C for 1 min and 60°C for 1 min. A final cycle was performed to determine the melting temperature of the test samples (from 50°C to 95°C).

The level of expression was determined by calculation of 2^−ΔΔCt^, where Δ*C*_t_ = (Vitiligo HERV *C*_t_ − Vitiligo GAPDH Endogenous Control *C*_t_) − (Average Δ*C*_t_ of all controls). The results were represented as fold change. Positive samples were confirmed with melting curve analysis. Importantly, positive and negative controls were added in every reaction.

### 2.4 Determination of systemic cytokine concentrations

The serum concentrations of the cytokines IL-1β, IL-6, IL-10, TNF-α, IFN-γ, IFN-λ1, and IFN-λ2/3 were determined using commercial ELISA kits (Thermo Fisher, Waltham, MA, USA) following the manufacturer’s instructions. The concentration of each cytokine was calculated through an appropriate standard curve that presented a correlation coefficient from 0.95 to 0.99, with the intra-assay coefficients of variance varying from 2.5% to 4% and those of the inter-assay from 8% to 10%.

### 2.5 Anti-HERV-W antibody detection

The HERV-W peptide TSSSPYQQ was constructed based on the HERV-W sequence (GeneBank accession no. AAI37382.1) and was used for the indirect ELISA. Briefly, the peptide was resuspended in 0.5 mg/ml of DMSO. For the ELISA, Stripwell 1 × 8 High-Binding Plates (Costar Corporation, Corning, NY, USA) were used. Approximately 10 μg/ml of the peptide was used to coat the plates with bicarbonate/carbonate buffer (pH 9.6) and incubated overnight in a humid chamber at 4°C. The plate was washed once with a washing solution composed of PBS containing 0.1% Tween-20. Subsequently, a blocking solution (PBS with 0.1% Tween-20 and 3% fetal bovine serum) was added at a volume of 200 µl per well and then incubated for 30 min at room temperature in a humid chamber. After incubation, the blocking solution was discarded and the plate was washed twice with the washing solution. Thereafter, 50 µl of the primary antibody (patient serum) diluted 1:500 in PBS was added, and the plate was incubated in a humid chamber at 37°C for 1 h. After incubation, the contents were discarded and the plate washed three times. Afterward, 50 µl of the secondary antibody (anti-human IgG conjugated with peroxidase) diluted 1:10,000 was added and incubated for 30 min at room temperature in a humid chamber. After this step, the contents were discarded and the plate washed three times. Finally, 50 µl of the substrate solution (ABTS; Sigma, St. Louis, MO, USA) prepared with hydrogen peroxide (H_2_O_2_) was added, and the plate was kept in the dark for 1 h. After incubation, 1 M HCl was added to stop the reaction, and absorbance was measured at 405 nm using a microplate reader (Loccus LMR-96, Loccus - Cotia Brazil).

### 2.6 Sample size calculation and statistical analysis

The sample size was initially considered in previous studies of our group (Morais et al., 2024; Rodrigues et al., 2019; Rangel et al., 2024; Silva et al., 2025). Considering that the majority of the individuals in previous studies presented basal expression levels of HERV, finding someone who expresses HERV is therefore nearly certain with minimal chances of mistakes. However, to guarantee a size effect of 80%, we used the *Z* approximation test by means, which resulted in 27 individuals per group with a confidence interval of 95%.

Significance analysis of the quantitative variables was initially performed for the normality distribution of the data using the Shapiro–Wilk test. As the data did not pass the normality test, we used the non-parametric Mann–Whitney test to compare the median scores between groups, the Krukal–Wallis test for intra-group analysis, and Spearman’s coefficient test for correlation analysis. All tests were conducted under the assumption of a type I error probability (alpha) of 5%.

## 3 Results

**Table 2** summarizes the demographic data and clinical findings of the volunteers enrolled in the study.

**Table 2:** Demographic data and clinical findings of the volunteers enrolled in the study.

Variable (*n*)	Vit (*n* = 30)		CTL (*n* = 30)		*p*
Sex	Female	Male	Female	Male	
	25	5	25	5	>0.999
Average age (years), SD	51 ± 14	52 ± 4	43 ± 9	54 ± 7	
Ethnic group					
Black	2 (6.6)	1 (3.3)	0	0	
Brown	15 (50)	1 (3.3)	13	4	0.259
White	8 (26.6)	3 (10)	12	1	
Skin phototype					
II	2 (6.6)	0	NA	NA	0.555
III	8 (26.6)	3 (10)			
IV	11 (36.6)	2 (6.6)			
V	4 (13.3)	0			
Age at vitiligo diagnosis (years), *n* (%)					
0–20	8 (26.6)	2 (6.6)	NA	NA	0.333
21–40	4 (13.3)	2 (6.6)			
41–60	13 (43.3)	1 (3.3)			
Vitiligo type, *n* (%)					
Vulgar	7 (23.3)	1 (3.3)	NA	NA	
Vulgar/acrofacial	11 (36.6)	3 (10)			0.587
Focal	5 (16.6)	0			
Universal	2 (6.6)	1 (3.3)			
Lesion evolution^a^, *n* (%)					
Improved	13 (43.3)	4 (13.3)	NA	NA	0.372
Stable	5 (16.6)	1 (3.3)			
Worsened	7 (23.3)	0 (0)			
Vitiligo extent score, *n* (%)					
	19 (63.4)	3 (10)	NA	NA	0.460
	6 (20)	2 (6.7)			
Comorbidities, *n* (%)					
Diabetes mellitus	4 (13.3)	2 (6.7)	1 (3.3)	1 (3.3)	0.521
Hypertension	4 (13.3)	3 (10)	1 (3.3)		

*Vit*, vitiligo group; *CTL*, control group; *SD*, standard deviation; *NA*, not applicable.

^a^ Evolution of the lesions was considered based on the medical history of the individuals in previous outpatient appointments.

**Figure 1:**
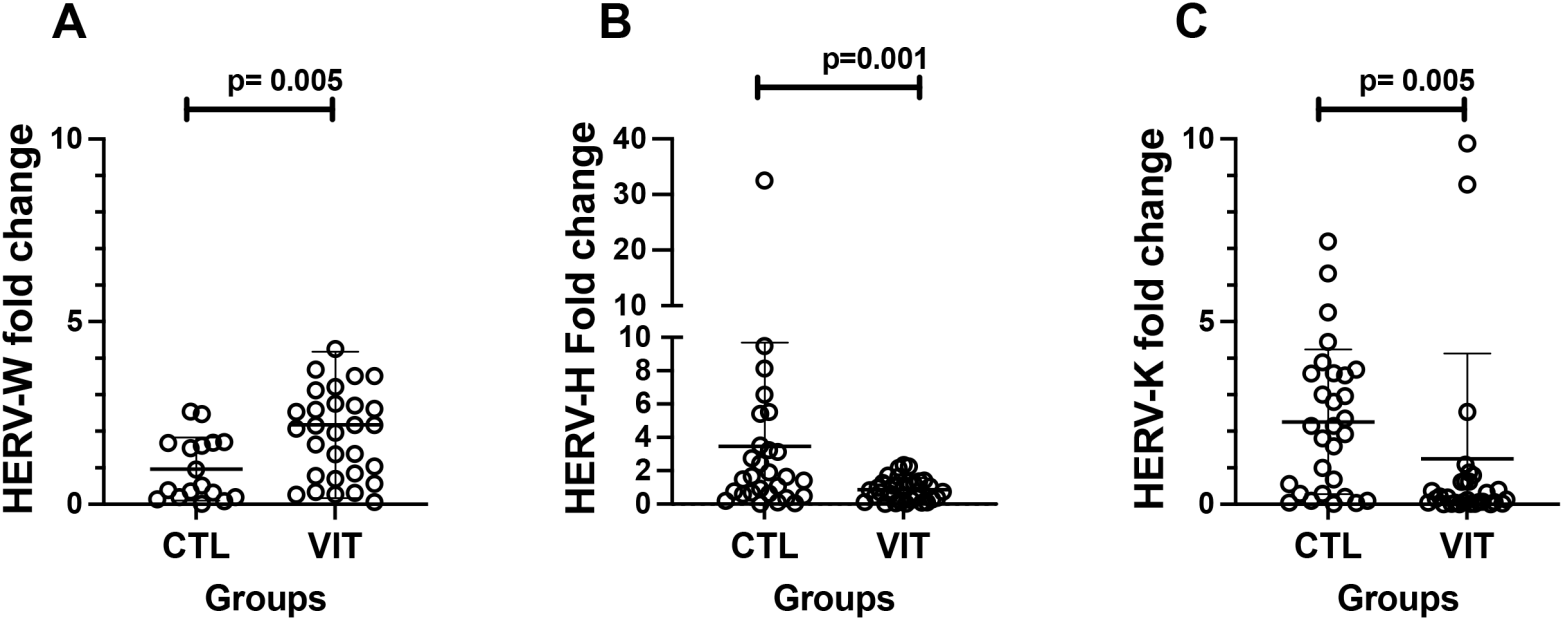
Relative expression of human endogenous retroviruses (HERVs) in individuals with vitiligo. HERV-W *env* **(A)** was upregulated in vitiligo patients (*p* = 0.005), whereas the expression levels of HERV-H *pol* **(B)** and HERV-K *gag* **(C)** were downregulated (Mann–Whitney test: *p* = 0.001 and *p* = 0.005, respectively). *Lines* show the median and confidence interval. *VIT*, vitiligo group; *CTL*, control group.

As expected, all individuals presented expression of all types of HERVs analyzed here. Interestingly, patients with vitiligo demonstrated upregulated expression levels of each HERV compared with the control group (**Figure 1**). Specifically, patients with vitiligo showed an approximately 2.5-fold upregulation of HERV-W *env* expression (**Figure 1A**), in conjunction with a downregulation of 3.5-fold in HERV-H *pol* (**Figure 1B**) and twofold in HERV-K *gag* (**Figure 1C**) expression compared with those found in healthy individuals (control group).

**Figure 2:**
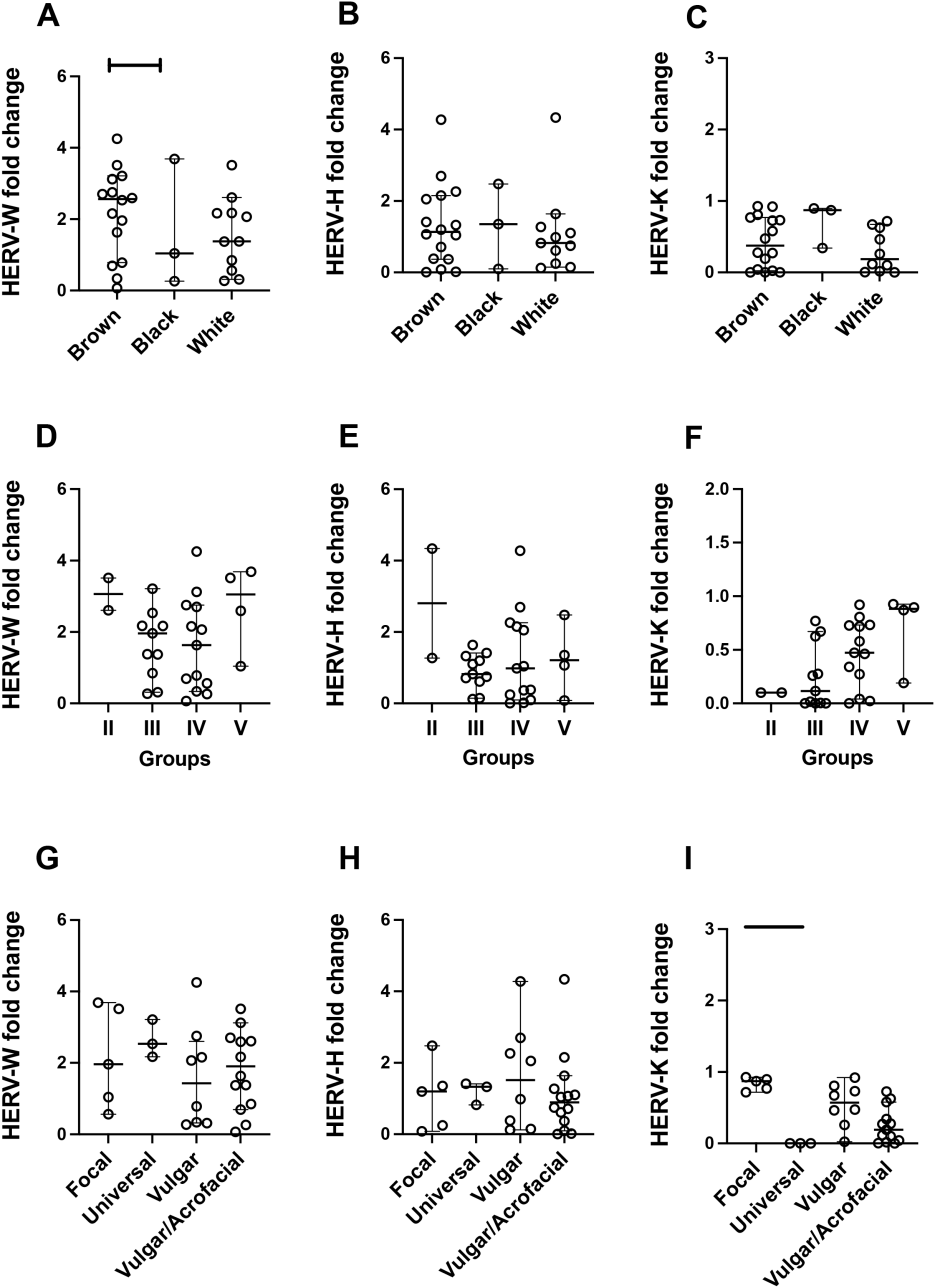
Profiles of human endogenous retrovirus (HERV)-W *env*, HERV-H *pol*, and HERV-K *gag* expression in vitiligo patients according to the ethnic characteristics **(A–C)**, the skin phototype **(D–F)**, and the vitiligo lesion type **(G–I)**. The only significance found was between the expression levels of HERV-W *env* in brown individuals and black individuals (*p* = 0.05) **(A)**, and *HERV-K* expression in universal individuals was lower than that in focal lesions (*p* < 0.01) **(I)**. No differences were observed in the other comparisons (Mann–Whitney test). *Lines* show the median and confidence interval.

**Figure 2** shows the results obtained in the analysis of the expression levels of the HERV types assessed here according to the ethnic group (**Figures 2A–C**), the skin phototype (**Figures 2D–F**), and the vitiligo lesion type (**Figures 2G–I**). As observed, brown people showed higher expression levels of HERV-W compared with black individuals (*p* = 0.05). No other significant differences were found.

**Figure 3:**
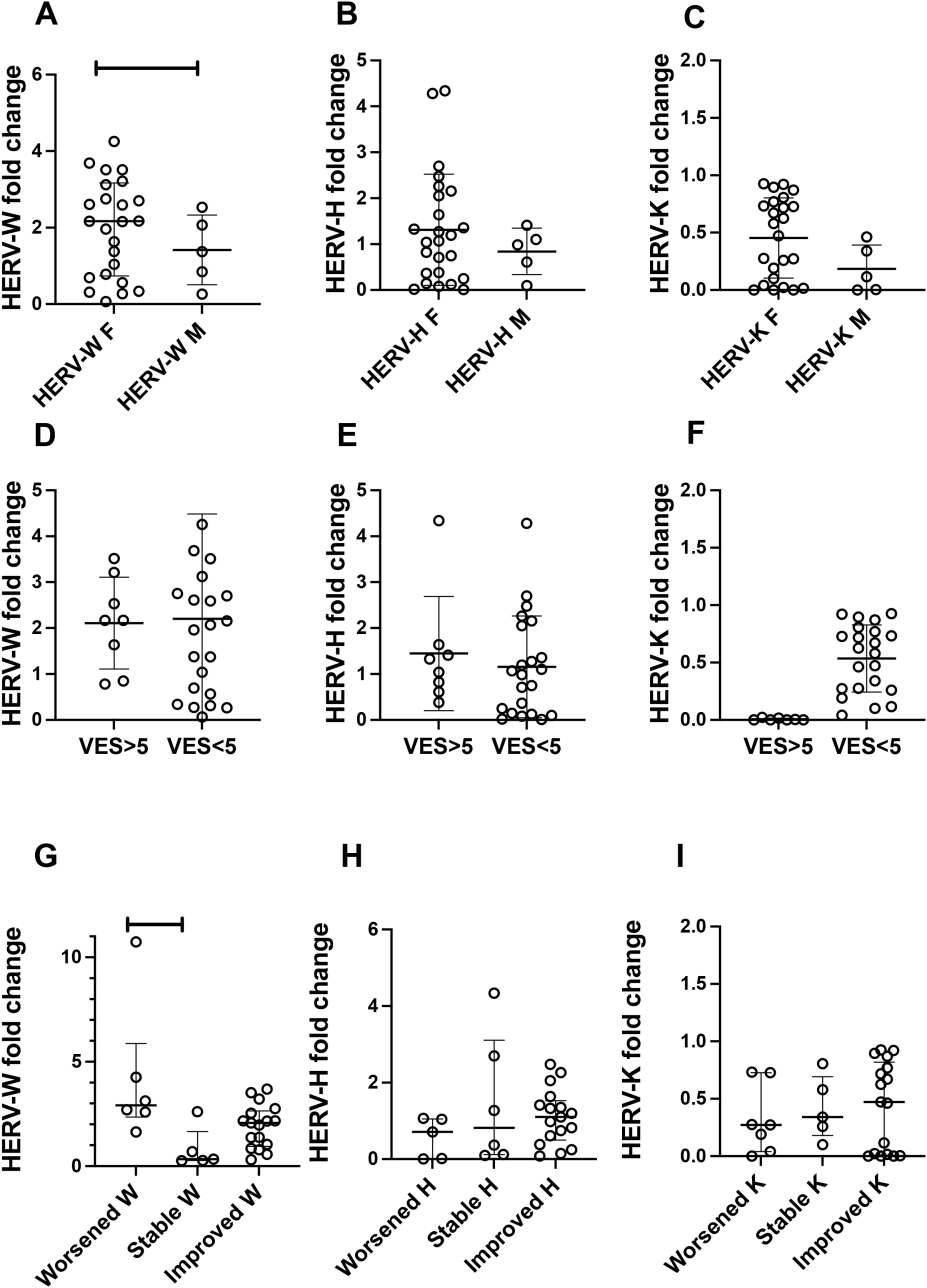
Profiles of human endogenous retrovirus (HERV)-W *env*, HERV-H *pol*, and HERV-K *gag* according to gender **(A–C)**, the vitiligo extent score (VES) **(D–F)**, and lesion conditions of vitiligo patients **(G–I)**. Mann–Whitney test was used. *HERV-K, HERV-W*, and *HERV-H F* indicate HERV family expression in female patients, while *HERV-K, HERV-W*, and *HERV-H M* denote HERV family expression in male patients. *VES*, vitiligo extent score. *p < 0.05, ****p < 0.0001 (Mann–Whitney test). *p*Lines show the median and confidence interval.

Subsequently, we sought to assess the expression levels of HERVs in relation to gender, the vitiligo extent score (VES), and the lesion condition. As shown in **Figure 3A**, it was observed that female vitiligo patients exhibited higher expression levels of HERV-W than the male patients. No differences were observed among the HERV expression levels according to the VES. Notably, patients whose lesions worsened during their most recent outpatient visits exhibited higher HERV-W than those with stable lesions (**Figure 3G**). Importantly, HERV-K presented low expression levels in all analyses, which may reveal a pattern of the expression of this retroelement in vitiligo.

**Figure 4:**
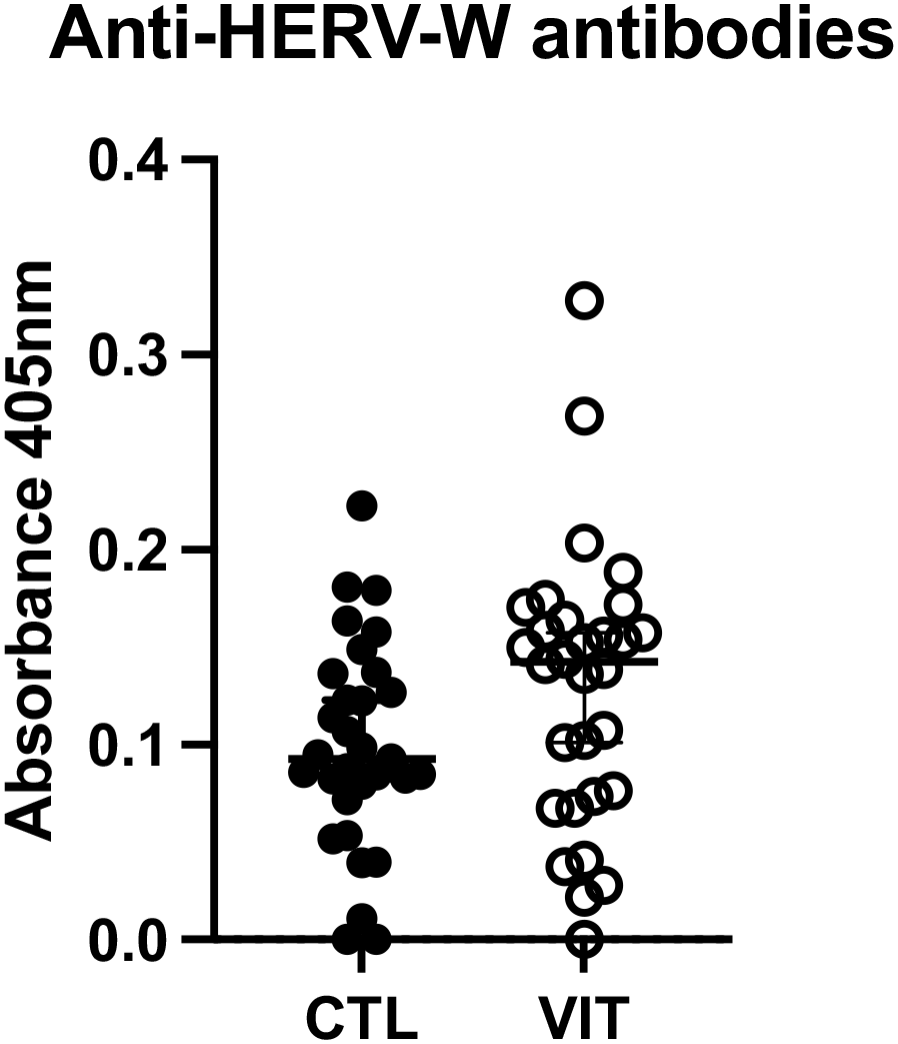
Anti-human endogenous retrovirus (HERV)-W *env* antibodies in vitiligo patients and healthy individuals assessed using ELISA. No difference was observed among groups (*p* = 0.0719). Mann–Whitney test was used. *Lines* show the median and confidence interval. *VIT*, vitiligo group; *CTL*, control group.

Following assessment of HERV expression, we analyzed whether the circulating levels of antibodies against HERV-W *env* in patients with vitiligo would be different compared with those in healthy controls. As shown in **Figure 4**, no difference was verified (*p* = 0.0719).

**Figure 5:**
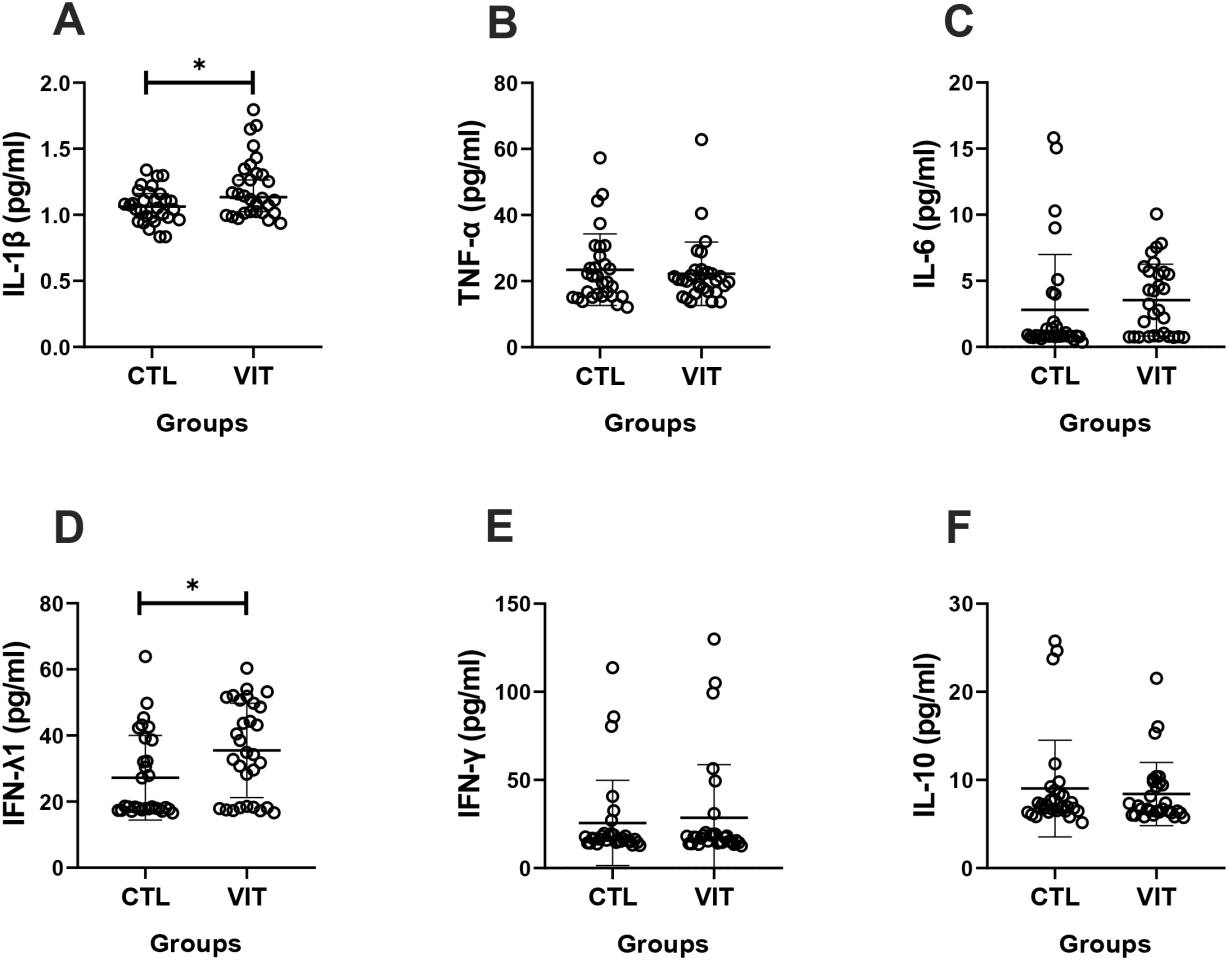
Serum concentrations of cytokines in vitiligo patients and healthy individuals. IL-1b**(A)**, TNF-a**(B)**, IL-6**(C)**, IFN-l1**(D)**, IFN-y**(E)** and IL-10**(F)** are demonstrated. Importantly, IL-1b**(A)** and IFN-l1**(D)** were demonstrated to be in significantly higher concentrations in vitiligo patients (p < 0.05). Mann–Whitney test was used. *Lines* show the median and confidence interval. *VIT*, vitiligo group; *p<0.05.

**Figure 5** illustrates the data obtained from the analysis of the systemic inflammatory status in vitiligo patients and the control group by evaluating the pro- and anti-inflammatory cytokine levels. As can be verified, patients with vitiligo presented higher circulating levels of IL-1β (**Figure 5A**) and IFN-λ1 (**Figure 5D**) compared with the control group.

**Figure 6:**
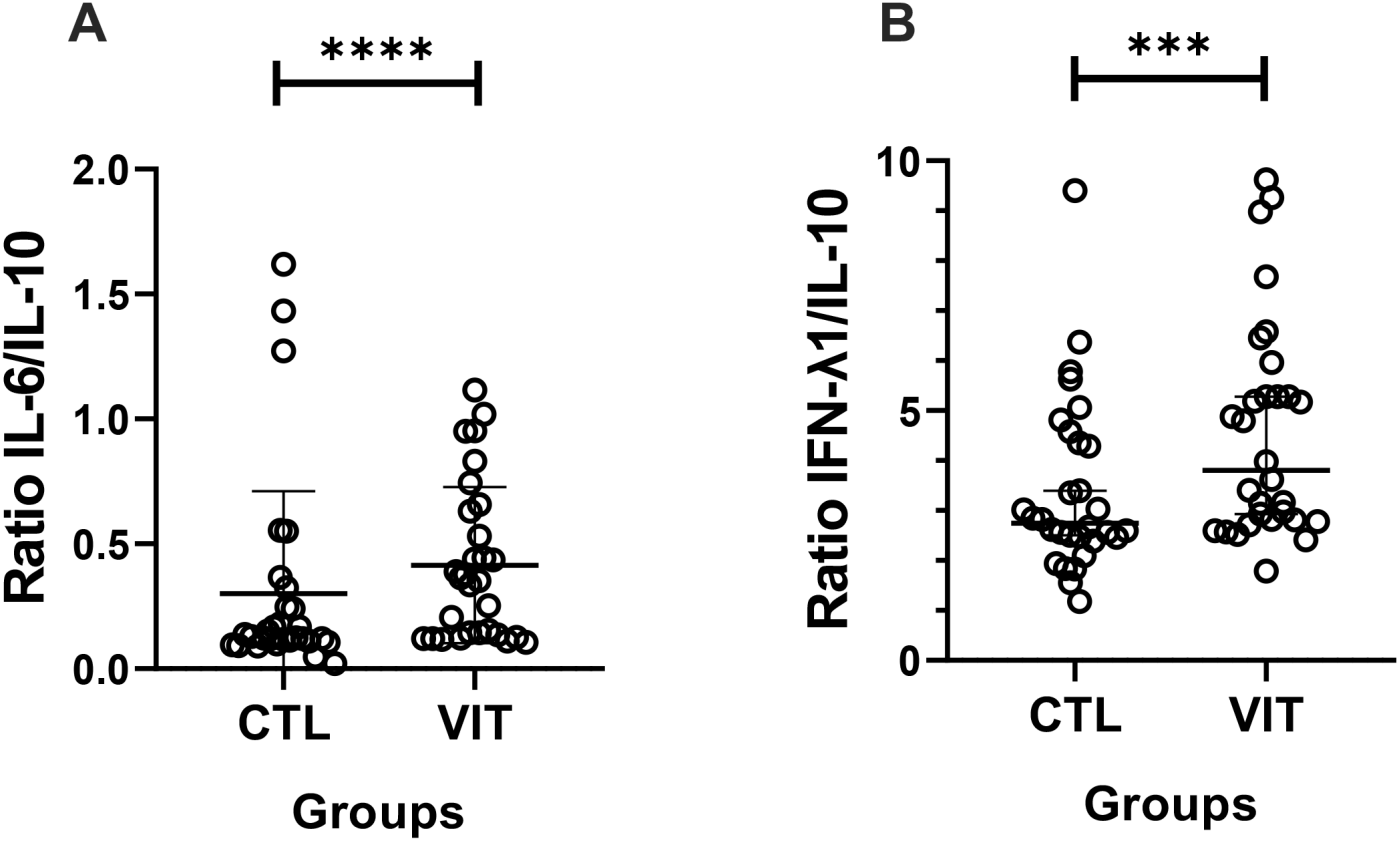
Pro-inflammatory and anti-inflammatory cytokine concentration ratios in the serum of vitiligo patients and healthy individuals. Vitiligo patients showed higher ratios of IL-6/IL-10 (*p* < 0.001) **(A)** and IFN-λ1/IL-10 (*p* < 0.01) **(B)**. Mann–Whitney test was used. *Lines* show the median and confidence interval. *VIT*, vitiligo group; *CTL*, control group. ***p<0.01 and ****p<0.001.

In order to better define the systemic inflammatory status in the volunteer groups, **Figure 6** shows the results obtained for the ratios between the pro-inflammatory cytokines and the anti-inflammatory IL-10. It was observed that patients with vitiligo presented higher IL-6/IL-10 (**Figure 6A**) and IFN-λ1/IL-10 ratios (**Figure 6B**) than healthy individuals.

**Figure 7:**
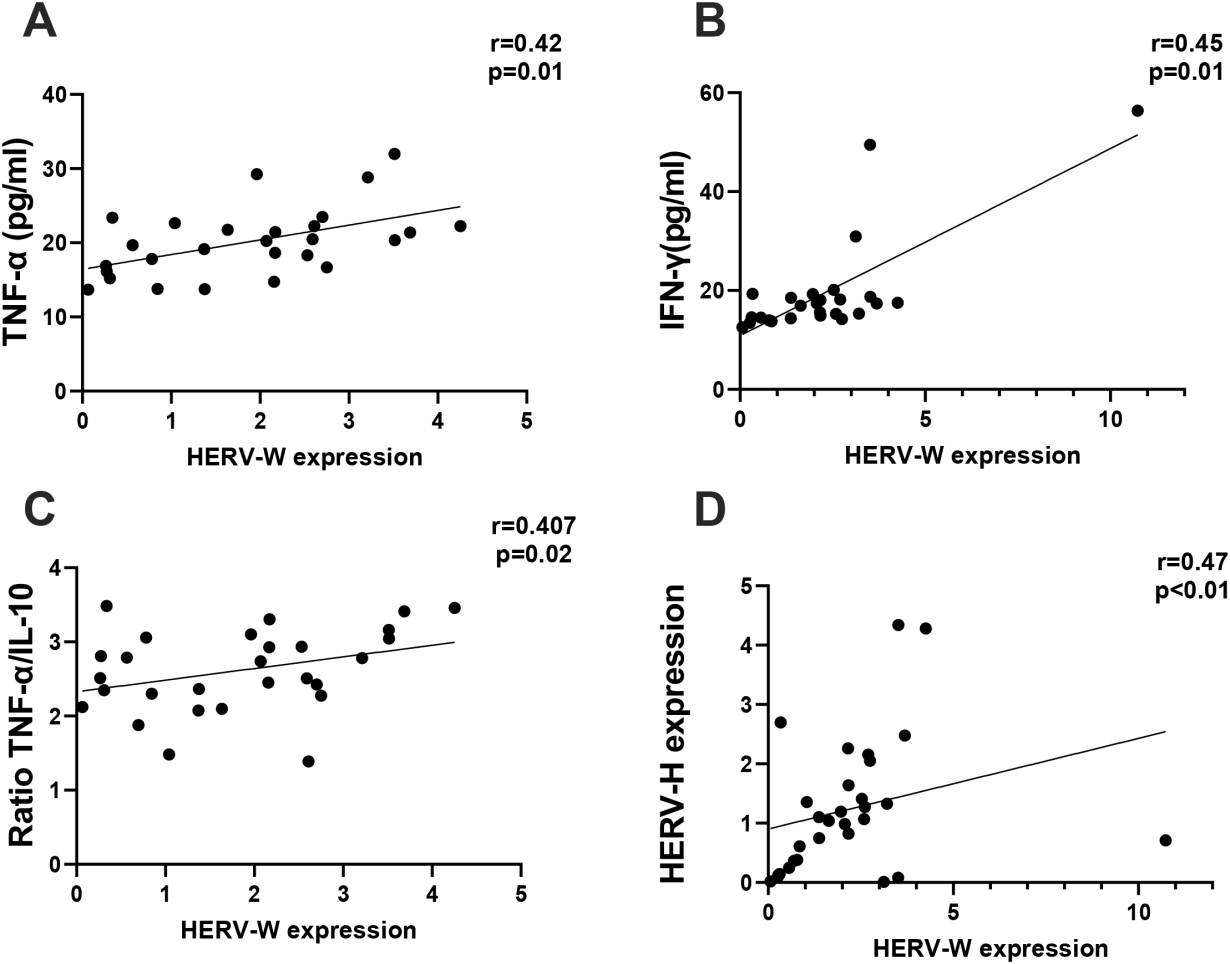
Spearman’s coefficient correlation analysis of the pro-inflammatory markers and the human endogenous retrovirus (HERV) expression levels. The analysis indicates a medium positive correlation between the expression level of HERV-W and TNF-α (*r* = 0.42, *p* = 0.01) **(A)** and IFN-γ (*r* = 0.45, *p* = 0.01) **(B)**, the ratio TNF-α/IL-10 (*r* = 0.40, *p* = 0.02) **(C)**, and the expression level of HERV-H (*r* = 0.47, *p* < 0.01) **(D)**.

Finally, we performed a correlation analysis between the expression levels of HERVs, the concentrations of cytokines, and the ratios of pro-inflammatory/anti-inflammatory cytokines. The results showed significant associations, which are presented in **Figure 7**. HERV-W expression was positively correlated not only with the circulating levels of TNF-α (**Figure 7**) and IFN-γ (**Figure 7B**) but also with the ratio of TNF-α/IL-10 (**Figure 7C**), as well as with the expression level of HERV-H (**Figure 7D**).

## 4 Discussion

Based on the data obtained in the present study, we were able to demonstrate that, while the expression levels of HERV-W *env* were higher in patients with vitiligo than in healthy individuals (~2.5-fold change, *p* = 0.005), the expression levels of both HERV-H *pol* and HERV-K *gag* were upregulated in the control group in comparison to vitiligo patients [~4-fold change (*p* = 0.001) and ~2-fold change (*p* = 0.005), respectively]. These findings, to our knowledge, represent the first description of the dynamics of HERV expression in human patients with vitiligo. Of note is that there is, until now, only a single study that focused on the potential role of HERVs in vitiligo using the animal model of the disease in Smyth line chickens. It was demonstrated that the expression of ERVs were associated with both the disease onset and the induction of the clinical manifestations of vitiligo (Sreekumar et al., 2000). These observations are in agreement with ours as we also identified an upregulation of HERV-W *env*. Importantly, HERV-W *env* is believed to induce an autoimmune response in some autoimmune diseases, such as MS, rheumatoid arthritis (RA), and systemic lupus erythematosus (SLE) (Mameli et al., 2015; Dolei, 2018; Rangel et al., 2024), as well as in inflammatory responses (Duperray et al., 2015) including neuropsychiatric disorders (Rangel et al., 2024). The potential role of HERV-W in autoimmune diseases has been investigated in depth in MS. Several pieces of evidence in the last 30 years have highlighted a key immunopathogenic role of this virus on this disease (Charvet et al., 2021; Rasmussen et al., 1995; Perron et al., 1997; Rolland et al., 2005; Rolland et al., 2006; Perron et al., 2013; Mameli et al., 2015; Tarlinton et al., 2020). Interestingly, the autoimmunity in vitiligo presents some possible mechanisms of immunopathogenesis related to cell damage, such as anti-melanocyte antibodies (Zhu et al., 2015) and lesions mediated by CD8^+^ T lymphocytes (Lang et al., 2001), in addition to the high concentrations of circulating pro-inflammatory cytokines such as IL-1β, IL-17, IFN-γ, and TGF-β (Speeckaert et al., 2017). Although an autoimmune response is evident in vitiligo, the factors that might contribute to triggering this condition or even stimulating the inflammatory response in vitiligo are still not fully understood. Therefore, our findings allow suggesting that HERV-W *env* may represent an additional piece to the puzzle of understanding the complex autoimmune/inflammatory responses associated with vitiligo. In contrast to the data on HERV-W expression, the same vitiligo patients showed a downregulation of the expression of HERV-K and HERV-H. According to the literature, HERV-K is believed to play a major role in many types of cancers (Dolci et al., 2025; Nguyen et al., 2025; Matteucci et al., 2025) and neurodegenerative manifestations in amyotrophic lateral sclerosis (ALS) (Pasternack et al., 2024). However, evidence associating HERV-K with autoimmune response is still incipient. Thus, our finding of the downregulation of HERV-K compared with those of the other HERV types assessed in patients with vitiligo is expected. Similarly to evidence in terms of HERV-K expression, HERV-H was also downregulated in patients with vitiligo. Several pieces of evidence in the literature point to the physiological role of HERV-H in the regulation of genes and the promotion of pluripotency (da Silva et al., 2024), in addition to its role in some cancers (Wentzensen et al., 2007; Liang et al., 2012) and, possibly, as regulators of the immune system (Bhatt et al., 2021).

Beyond these findings, interestingly, brown people presented higher expression levels of HERV-W *env* than white and black people. Although there are only a few studies aiming to understand the dynamics of HERV expression among distinct ethnic groups, previous findings revealed that HERV expression among ethnic groups may differ (Alldredge et al., 2023; Tarlinton et al., 2020). In addition, the observation that female vitiligo patients presented higher expression levels of HERV-W *env* than the male group is intriguing and will be further addressed here. Interestingly, the predominance of women affected by vitiligo is not observed in this autoimmune disease (Haulrig et al., 2024). However, in other autoimmune diseases, such MS, the occurrence is more prevalent in women, and both healthy subjects and women with MS present higher HERV DNA proviral copy number than men (Garcia-Montojo et al., 2013). This might be explained by the fact that the X chromosome is among those with the highest number of HERV copies in the human genome (da Silva et al., 2024). In light of these observations, it is feasible to presume that women might present higher expression levels of HERV than men, which is in agreement with what was observed here. However, this is still an uncertain observation. A previous study did not observe any differences in the expression levels of HERVs between men and women (Balestrieri et al., 2015). This might reveal a particular profile of HERV-W *env* expression in vitiligo, where the expression dynamics behaves differently and appears to be more active in women. Although still not fully understood, this may suggest that HERV expression may be increased in women affected by some autoimmune diseases.

It is worth noting that the discrepancy in the expression levels of the HERV families found here could also be associated with the clinical conditions of the patients. Thus, it is of utmost importance to highlight that patients who presented worsening in skin lesions presented considerably higher expression levels of HERV-W *env* than those who presented stable lesions. This finding is intriguing and may reveal some possible conditions: i) the higher expression of HERV-W *env* may be associated with a clinical condition driven by local inflammation or ii) the high expression of HERV-W *env* may be associated with the persistent ongoing lesion in vitiligo. Corroborating these observations, it was reported in other autoimmune disease models that the expression level of HERV-W was also associated with worse clinical conditions (Nali et al., 2022; Sotgiu et al., 2010). Moreover, it is well established that a pro-inflammatory condition may promote HERV expression locally and systematically, and HERV-W *env* may induce inflammatory response in many conditions (Garza et al., 2023; Tamouza et al., 2023; Wang et al., 2023; Lima-Junior et al., 2021; Gruchot et al., 2023). Although we could only determine a possible association, a possible scenario could feasibly occur where both inflammation and HERV are stimulated, promoting a possible “snowball” effect that could drive to lesions and the maintenance of the worsening condition of the lesions. Based on these pieces of information, our results allow suggesting that HERV-W *env* expression can play a corollary role in patients with vitiligo who present persistent tissue damage.

Altogether, our findings on the dynamics of HERV expression might reveal an interesting profile of both the upregulation of HERV-W and the downregulation of other HERV families. Furthermore, we found moderate correlations between the expression levels of HERV-W and HERV-H, which may reveal a scenario that the expression of the HERV family may be distinct in different pathological processes, such as autoimmune response and cancer, and may be a compensatory role of distinct HERV expression. In addition, it is of utmost importance to highlight that this novel report may be relevant to: i) the use of HERV-W *env* expression as a possible biomarker for both the presence and the severity of the disease and ii) as a potential target for experimental analysis and possibly a target for possible treatment of the disease. In addition, a downregulated expression of HERV-H may reveal an interesting scenario: as mentioned, this HERV role may be linked to a regulation of the immune system acting as an immune checkpoint (Bhatt et al., 2021), and a high expression of this HERV may be associated with cancer types (Wentzensen et al., 2007; Liang et al., 2012). Conversely, in infection models such as viral bronchiolitis, the expression of HERV-H was also downregulated (Tovo et al., 2023a). Physiologically, during pregnancy, HERV-H may also be downregulated even in mothers affected by MS (Tovo et al., 2023b). In this sense, the downregulation of HERV-H should be further explored in order to reveal a possible role in this and in other autoimmune diseases, as well as its involvement in the expression of other HERVs.

In addition to the assessment of the expression of HERVs, we also aimed to determine the circulating levels of the anti-HERV-W *env* antibodies using an “in-house” ELISA. It is important to mention that the lack of a significant statistical difference in this evaluation between the volunteer groups could be explained by the fact that none of the patients reported active vitiligo lesions, which could ultimately impact their levels. Taken together, these data can still suggest a possible role of the anti-HERV-antibodies in the immunopathogenesis of vitiligo.

As previously mentioned, the assessment of the systemic inflammatory status in the volunteer groups was also another goal of this study. In this respect, patients with vitiligo presented higher circulating levels of IL-1β and IFN-λ1 compared with healthy individuals, which indicates the presence of a systemic status toward a pro-inflammatory profile in these patients. Regarding IFN-λ1, until now, this is the first study reporting a significant increase in their systemic levels in patients with vitiligo compared with healthy individuals. In fact, this finding may reveal a possible intriguing role of this cytokine in the context of vitiligo as it is broadly known that IFN-λ1 has a pivotal role in the antiviral response of both DNA (Lopušná et al., 2014) and RNA viruses (Mallampalli et al., 2021; Savino et al., 2025), including retroviruses (Guo et al., 2018; Mozhgani et al., 2018). Thus, the elevation in their levels can putatively suggest a possible mechanism of response against HERVs. As appealing as this role can be, it is important to cite that a previous study reported that IFN-λ1 can also impact on the JAK/STAT kinase pathway (Tang et al., 2025), which, consequently, may contribute to the impairment of CD8^+^ T homoeostasis (Fortelny et al., 2024), leading to the recruitment of CD8^+^ T lymphocytes to the skin, thus favoring an autoimmune response mediated by these cells, a common characteristic found in vitiligo lesions (Pathak et al., 2024; Luiten et al., 2009). Corroborating our observation, increased IFN-λ1 levels both in serum and tissue have been verified in individuals with different autoimmune diseases, particularly rheumatic autoimmune diseases (Goel et al., 2021). In agreement with the literature, an increase in the systemic IL-1β levels was evidenced in patients with vitiligo (Tomaszewska et al., 2020). It was suggested that this well-known pro-inflammatory cytokine might be involved in the progression of vitiligo, not only by favoring a T helper 1 (Th1) immune response but also in conjunction with other pro-inflammatory cytokines by inducing the inducible nitric oxide synthase (iNOS), a cytotoxic effector molecule (Ramirez-Ramirez et al., 2013). Interestingly, elevations in the systemic levels of IL-1β, as well as IL-6 and TNF-α, are promoted by IFN-γ, which, when combined, contribute to local and systemic inflammation in patients with vitiligo (Paganelli et al., 2025). Based on this information, although it was expected that circulating TNF-α, IL-6, and mainly IFN-γ levels would be increased in the vitiligo group, as observed with IL-1β, according to the literature, variations in some IFN-γ signaling genes can affect the risk of vitiligo (Upadhya et al., 2025). In addition, the fact that these volunteers were in treatment may have putatively led to the absence of significant differences between volunteer groups, as it is broadly accepted that higher IFN-γ levels are found in both pathogenesis and active vitiligo (Liu et al., 2025). Therefore, despite the treatment being able to regulate some of the cytokines frequently found in vitiligo, the higher circulating levels of IFN-λ1 and IL-1β in the vitiligo group reinforced the persistent pro-inflammatory status in this disease.

In addition to these results, it is worth mentioning that, although analysis of the cytokine levels was performed in isolation, each day, it was highlighted that the ratio between pro- and anti-inflammatory cytokines can provide an accurate measure to identify the balance/imbalance of the inflammatory process (Carvalho et al., 2024; Santos et al., 2023). Based on this, the higher ratios of IL-6/IL-10 and IFN-λ1/IL-10 in patients with vitiligo compared with the control group are interesting and can reinforce the suggestion of the presence of a systemic pro-inflammatory status in these individuals. As previously mentioned, elevated serum levels of IL-6 have been documented in patients with vitiligo (Bhatt et al., 2021), and it was suggested that the cytokine IL-6 presents an autocrine loop that, in conjunction with a synergistic action of IFN-γ, putatively promotes melanocyte apoptosis (Alldredge et al., 2023), thus favoring vitiligo progression. Besides these interesting manifestations, in order to improve our understanding of the impact of HERV expression in the context of vitiligo, we also carried out correlation analyses. According to the results, the expression level of HERV-W *env* was positively associated not only with the circulating levels of TNF-α and IFN-γ but also with the TNF-α/IL-10 ratio. Although the systemic levels of these pro-inflammatory cytokines did not show differences between volunteer groups, their positive association with HERV-W expression corroborates the literature (Rangel et al., 2022) as it demonstrates that HERV is closely associated with inflammatory responses. Particularly in the vitiligo scenario, these observations, together with the positive association between HERV-W expression and TNF-α/IL-10 ratio, are very interesting as these cytokines are believed to play a role in the autoimmunopathological response in vitiligo (Belpaire et al., 2025; Bhuyan et al., 2025; Ferreira et al., 2025). Considering these findings, we may putatively suggest that HERV expression has a prominent involvement toward the pro-inflammatory status in patients with vitiligo.

Lastly, despite the exploratory nature of the present study, we were able to present some novelties in relation to a distinct dynamic of HERV expression, as well as a possible and interesting involvement of HERV expression in the systemic inflammatory status of patients with vitiligo. However, some limitations have to be mentioned, including the lack of the inclusion of patients who were undergoing active vitiligo lesions, which allows determining the possible role of anti-HERV antibodies as pivotal players in the autoimmune response by molecular mimicry as HERVs share similarity with other self-proteins (do Olival et al., 2013; Ramasamy et al., 2017). Moreover, the associations found here should be further investigated in order to understand the possible causality of HERVs on the immunopathogenesis of the disease. Another limitation that should be addressed is the fact that we only had a small sample in the subgroup analysis of the clinical findings and HERV expression; however, the statistical analysis revealed promising findings that should be further investigated in future studies.

## 5 Conclusion

Our findings showed the dynamics of HERV expression in vitiligo and may pave the way for understanding the possible pivotal role of HERVs, in particular HERV-W *env*, on the immunopathogenesis of vitiligo. The low expression levels of HERV-K *gag* and HERV-H *pol* added to the increased levels of HERV-W and the systemic inflammatory profile could indicate that HERV-W might contribute to the systemic pro-inflammatory milieu characteristic of the disease. Furthermore, the expression pattern of HERV-W *env* highlights its potential utility as a biomarker for disease presence, activity, or progression, particularly in identifying patients with more extensive or worsening lesions. This deserves further investigation. Altogether, these results strengthen the evidence supporting the involvement of HERV elements in autoimmune diseases, and now in vitiligo, and open new avenues for research aimed at elucidating their mechanistic roles and therapeutic potential in vitiligo.

## Data Availability

In accordance with ethical and confidentiality requirements of the local ethical comittee regiments, the datasets supporting the findings of this study are not publicly available but can be obtained from the corresponding author upon request.

## References

[B1] Alldredge J. Kumar V. Nguyen J. Sanders B. E. Gomez K. Jayachandran K. . (2023). Endogenous retrovirus RNA expression differences between race, stage and HPV status offer improved prognostication among women with cervical cancer. Int. J. Mol. Sci. 24. doi: 10.3390/IJMS24021492, PMID: 36675007 PMC9864224

[B2] Balestrieri E. Pica F. Matteucci C. Zenobi R. Sorrentino R. Argaw-Denboba A. . (2015). Transcriptional activity of human endogenous retroviruses in human peripheral blood mononuclear cells. Biomed Res. Int. 2015, 164529. doi: 10.1155/2015/164529, PMID: 25734056 PMC4334862

[B3] Belpaire A. Demeyer A. Berrevoet D. Van Nieuwerburgh F. Van Caelenberg E. Papageorgiou T. . (2025). Downregulated aryl hydrocarbon receptor expression is linked with increased IFN-γ production and impaired immune checkpoint upregulation in vitiligo. J. Invest. Dermatol. doi: 10.1016/j.jid.2025.07.027, PMID: 40816656

[B4] Bhatt R. S. Berjis A. Konge J. C. Mahoney K. M. Klee A. N. Freeman S. S. . (2021). KIR3DL3 is an inhibitory receptor for HHLA2 that mediates an alternative immunoinhibitory pathway to PD1. Cancer Immunol. Res. 9, 156–169. doi: 10.1158/2326-6066.CIR-20-0315, PMID: 33229411 PMC8284010

[B5] Bhuyan G. Narah M. Khound R. Dutta T. (2025). Evaluating interferon γ to interleukin 10 ratio as a biomarker for stability and severity in vitiligo: A clinical and histopathologic correlation. Arch. Pathol. Lab. Med. doi: 10.5858/ARPA.2025-0099-OA, PMID: 40490250

[B6] Carvalho R. L. Brito T. R. P. Amaral J. B. Monteiro F. R. Lima D. B. Pereira T. A. M. . (2024). Unraveling the interaction between inflammation and the cardiometabolic index in older men: A pilot study. Nutrients 16. doi: 10.3390/NU16152529, PMID: 39125408 PMC11313730

[B7] Charvet B. Pierquin J. Brunel J. Gorter R. Quétard C. Horvat B. . (2021). Human endogenous retrovirus type W envelope from multiple sclerosis demyelinating lesions shows unique solubility and antigenic characteristics. Virol. Sin. doi: 10.1007/s12250-021-00372-0, PMID: 33770381 PMC8558138

[B8] Chen J. Li S. Li C. (2021). Mechanisms of melanocyte death in vitiligo. Med. Res. Rev. 41, 1138–1166. doi: 10.1002/med.21754, PMID: 33200838 PMC7983894

[B9] da Silva A. L. Guedes B. L. M. Santos S. N. Correa G. F. Nardy A. Nali L. H. d. S. . (2024). Beyond pathogens: the intriguing genetic legacy of endogenous retroviruses in host physiology. Front. Cell. Infect. Microbiol. 14. doi: 10.3389/fcimb.2024.1379962, PMID: 38655281 PMC11035796

[B10] de Jonge H. J. M. Fehrmann R. S. N. de Bont E. S. J. M. Hofstra R. M. W. Gerbens F. Kamps W. A. . (2007). Evidence based selection of housekeeping genes. PLoS One 2, e898. doi: 10.1371/journal.pone.0000898, PMID: 17878933 PMC1976390

[B11] Dolci M. Civettini I. Bagnoli P. F. Toumi W. Signorini L. Crocchiolo R. . (2025). Expression of human endogenous retrovirus env gene product is a hallmark of sidedness in operable colorectal cancer. Oncology. doi: 10.1159/000543099, PMID: 40068636

[B12] Dolei A. (2018). The aliens inside us: HERV-W endogenous retroviruses and multiple sclerosis. Mult. Scler. 24, 42–47. doi: 10.1177/1352458517737370, PMID: 29307292

[B13] do Olival G. S. Faria T. S. Nali L. H. S. de Oliveira A. C. P. Casseb J. Vidal J. E. . (2013). Genomic analysis of ERVWE2 locus in patients with multiple sclerosis: absence of genetic association but potential role of human endogenous retrovirus type W elements in molecular mimicry with myelin antigen. Front. Microbiol. 4, 172. doi: 10.3389/fmicb.2013.00172, PMID: 23805135 PMC3693062

[B14] Duperray A. Barbe D. Raguenez G. Weksler B. B. Romero I. A. Couraud P. O. . (2015). Inflammatory response of endothelial cells to a human endogenous retrovirus associated with multiple sclerosis is mediated by TLR4. Int. Immunol. 27, 545–553. doi: 10.1093/intimm/dxv025, PMID: 25957268 PMC4625887

[B15] Ezzedine K. Eleftheriadou V. Whitton M. van Geel N. (2015). Vitiligo. Lancet 386, 74–84. doi: 10.1016/S0140-6736(14)60763-7, PMID: 25596811

[B16] Ezzedine K. Lim H. W. Suzuki T. Katayama I. Hamzavi I. Lan C. C. E. . (2012). Revised classification/nomenclature of vitiligo and related issues: the Vitiligo Global Issues Consensus Conference. Pigment Cell Melanoma Res. 25, E1. doi: 10.1111/j.1755-148X.2012.00997.x, PMID: 22417114 PMC3511780

[B17] Ferreira C. King B. Torres T. (2025). JAK inhibitors for the treatment of vitiligo: current evidence and emerging therapeutic potential. Drugs. doi: 10.1007/s40265-025-02246-1, PMID: 40996476

[B18] Filipponi D. Muller J. Emelyanov A. Bulavin D. V. (2013). Wip1 controls global heterochromatin silencing via ATM/BRCA1-dependent DNA methylation. Cancer Cell 24, 528–541. doi: 10.1016/j.ccr.2013.08.022, PMID: 24135283

[B19] Fortelny N. Farlik M. Fife V. Gorki A. D. Lassnig C. Maurer B. . (2024). JAK-STAT signaling maintains homeostasis in T cells and macrophages. Nat. Immunol. 25, 847. doi: 10.1038/s41590-024-01804-1, PMID: 38658806 PMC11065702

[B20] Fukuda K. (2022). Networks of CD8+ T cell response activation in melanoma and vitiligo. Front. Immunol. 13. doi: 10.3389/fimmu.2022.866703, PMID: 35432377 PMC9011047

[B21] Garcia-Montojo M. Dominguez-Mozo M. Arias-Leal A. Garcia-Martinez Á. De las Heras V. Casanova I. . (2013). The DNA copy number of human endogenous retrovirus-W (MSRV-type) is increased in multiple sclerosis patients and is influenced by gender and disease severity. PLoS One 8, e53623. doi: 10.1371/journal.pone.0053623, PMID: 23308264 PMC3538585

[B22] Garza R. Sharma Y. Atacho D. A. M. Thiruvalluvan A. Abu Hamdeh S. Jönsson M. E. . (2023). Single-cell transcriptomics of human traumatic brain injury reveals activation of endogenous retroviruses in oligodendroglia. Cell Rep. 42. doi: 10.1016/j.celrep.2023.113395, PMID: 37967557

[B23] Goel R. R. Kotenko S. V. Kaplan M. J. (2021). Interferon lambda in inflammation and autoimmune rheumatic diseases. Nat. Rev. Rheumatol. 17, 349–362. doi: 10.1038/s41584-021-00606-1, PMID: 33907323 PMC8077192

[B24] Griffiths D. J. (2001). Endogenous retroviruses in the human genome sequence. Genome Biol. 2, REVIEWS1017. Available online at: http://www.pubmedcentral.nih.gov/articlerender.fcgi?artid=138943&tool=pmcentrez&rendertype=abstract (Accessed June 9, 2015). 11423012 10.1186/gb-2001-2-6-reviews1017 PMC138943

[B25] Grimes P. E. Miller M. M. (2018). Vitiligo: Patient stories, self-esteem, and the psychological burden of disease. Int. J. Womens Dermatol. 4, 32–37. doi: 10.1016/j.ijwd.2017.11.005, PMID: 29872674 PMC5986114

[B26] Gruchot J. Herrero F. Weber-Stadlbauer U. Meyer U. Küry P. (2023). Interplay between activation of endogenous retroviruses and inflammation as common pathogenic mechanism in neurological and psychiatric disorders. Brain Behav. Immun. 107, 242–252. doi: 10.1016/j.bbi.2022.10.007, PMID: 36270439

[B27] Guo L. Xu X. Q. Zhou L. Zhou R. H. Wang X. Li J. L. . (2018). Human intestinal epithelial cells release antiviral factors that inhibit HIV infection of macrophages. Front. Immunol. 9. doi: 10.3389/fimmu.2018.00247, PMID: 29515574 PMC5825896

[B28] Guo Y. Yang C. Liu Y. Li T. Li H. Han J. . (2022). High expression of HERV-K (HML-2) might stimulate interferon in COVID-19 patients. Viruses 14, 996. doi: 10.3390/v14050996, PMID: 35632738 PMC9143815

[B29] Haulrig M. B. Al-Sofi R. Baskaran S. Bergmann M. S. Løvendorf M. Dyring-Andersen B. . (2024). The global epidemiology of vitiligo: A systematic review and meta-analysis of the incidence and prevalence. JEADV Clin. Pract. 3, 1410–1419. doi: 10.1002/jvc2.526, PMID: 41925078

[B30] Lang K. S. Caroli C. C. Muhm A. Wernet D. Moris A. Schittek B. . (2001). HLA-A2 restricted, melanocyte-specific CD8+ T lymphocytes detected in vitiligo patients are related to disease activity and are predominantly directed against MelanA/MART1. J. Invest. Dermatol. 116, 891–897. doi: 10.1046/j.1523-1747.2001.01363.x, PMID: 11407977

[B31] Le Poole I. C. Wañkowicz-Kaliñska A. Van Den Wijngaard R. M. J. G. J. Nickoloff B. J. Das P. K. Bos J. D. . (2004). Autoimmune aspects of depigmentation in vitiligo. J. Invest. Dermatol. Symp. Proc. 9, 68–72. doi: 10.1111/j.1087-0024.2004.00825.x, PMID: 14870989

[B32] Li F. Nellåker C. Yolken R. H. Karlsson H. (2011). A systematic evaluation of expression of HERV-W elements; influence of genomic context, viral structure and orientation. BMC Genomics 12, 22. doi: 10.1186/1471-2164-12-22, PMID: 21226900 PMC3031232

[B33] Liang Q. Xu Z. Xu R. Wu L. Zheng S. (2012). Expression patterns of non-coding spliced transcripts from human endogenous retrovirus HERV-H elements in colon cancer. PLoS One 7, e29950. doi: 10.1371/journal.pone.0029950, PMID: 22238681 PMC3253121

[B34] Lima-Junior D. S. Krishnamurthy S. R. Bouladoux N. Collins N. Han S. J. Chen E. Y. . (2021). Endogenous retroviruses promote homeostatic and inflammatory responses to the microbiota. Cell 184, 3794–3811.e19. doi: 10.1016/j.cell.2021.05.020, PMID: 34166614 PMC8381240

[B35] Liu B. Shen J. Li J. Tian B. Zhou B. Gui J. . (2025). Candidate approaches for predicting vitiligo recurrence: an effective model and biomarkers. Front. Immunol. 16. doi: 10.3389/fimmu.2025.1468665, PMID: 39981245 PMC11839629

[B36] Lopušná K. Režuchová I. Kabát P. Kúdelová M. (2014). Interferon lambda induces antiviral response to herpes simplex virus 1 infection. Acta Virol. 58, 325–332. doi: 10.4149/av_2014_03_325, PMID: 25518713

[B37] Luiten R. M. Van Den Boorn J. G. Konijnenberg D. Dellemijn T. A. M. Van Der Veen J. P. W. Bos J. D. . (2009). Autoimmune destruction of skin melanocytes by perilesional T cells from vitiligo patients. J. Invest. Dermatol. 129, 2220–2232. doi: 10.1038/jid.2009.32, PMID: 19242513

[B38] Mallampalli R. K. Adair J. Elhance A. Farkas D. Chafin L. Long M. E. . (2021). Interferon lambda signaling in macrophages is necessary for the antiviral response to influenza. Front. Immunol. 12. doi: 10.3389/fimmu.2021.735576, PMID: 34899695 PMC8655102

[B39] Mameli G. Astone V. Arru G. Marconi S. Lovato L. Serra C. . (2007). Brains and peripheral blood mononuclear cells of multiple sclerosis (MS) patients hyperexpress MS-associated retrovirus/HERV-W endogenous retrovirus, but not Human herpesvirus 6. J. Gen. Virol. 88, 264–274. doi: 10.1099/vir.0.81890-0, PMID: 17170460

[B40] Mameli G. Cossu D. Cocco E. Frau J. Marrosu M. G. Niegowska M. . (2015). Epitopes of HERV-Wenv induce antigen-specific humoral immunity in multiple sclerosis patients. J. Neuroimmunol. 280, 66–68. doi: 10.1016/j.jneuroim.2015.03.003, PMID: 25773158

[B41] Mameli G. Poddighe L. Mei A. Uleri E. Sotgiu S. Serra C. . (2012). Expression and activation by Epstein Barr virus of human endogenous retroviruses-W in blood cells and astrocytes: inference for multiple sclerosis. PLoS One 7, e44991. doi: 10.1371/journal.pone.0044991, PMID: 23028727 PMC3459916

[B42] Matteucci C. Petrone V. Giovinazzo A. Laureana R. Postorino M. Pupo L. . (2025). Expression of HERV-K and embryonic genes in chronic lymphocytic leukemia and their association with therapy regimens. Blood Adv. 9, 4265–4278. doi: 10.1182/bloodadvances.2024014181, PMID: 40493879 PMC12395068

[B43] Medstrand P. Landry J. R. Mager D. L. (2001). Long terminal repeats are used as alternative promoters for the endothelin B receptor and apolipoprotein C-I genes in humans. J. Biol. Chem. 276, 1896–1903. doi: 10.1074/jbc.M006557200, PMID: 11054415

[B44] Mi S. Lee X. Li X. Veldman G. M. Finnerty H. Racie L. . (2000). Syncytin is a captive retroviral envelope protein involved in human placental morphogenesis. Nature 403, 785–789. doi: 10.1038/35001608, PMID: 10693809

[B45] Morais L. V. dos Santos S. N. Gomes T. H. Romano C. M. Colombo-Souza P. Amaral J. B. . (2024). Acute strength exercise training impacts differently the HERV-W expression and inflammatory biomarkers in resistance exercise training individuals. PLoS One 19. doi: 10.1371/journal.pone.0303798, PMID: 38753716 PMC11098355

[B46] Mozhgani S. H. Jahantigh H. R. Rafatpanah H. Valizadeh N. Mohammadi A. Basharkhah S. . (2018). Interferon lambda family along with HTLV-1 proviral load, tax, and HBZ implicated in the pathogenesis of myelopathy/tropical spastic paraparesis. Neurodegener. Dis. 18, 150–155. doi: 10.1159/000490058, PMID: 29990995

[B47] Nali L. H. S. Olival G. S. Montenegro H. da Silva I. T. Dias-Neto E. Naya H. . (2022). Human endogenous retrovirus and multiple sclerosis: A review and transcriptome findings. Mult. Scler. Relat. Disord. 57. doi: 10.1016/j.msard.2021.103383, PMID: 34922254

[B48] Nellåker C. Yao Y. Jones-Brando L. Mallet F. Yolken R. H. Karlsson H. (2006). Transactivation of elements in the human endogenous retrovirus W family by viral infection. Retrovirology 3, 44. doi: 10.1186/1742-4690-3-44, PMID: 16822326 PMC1539011

[B49] Nguyen Q. T. T. Shin S. G. Nguyen T. T. T. Lee K. Y. McClelland M. Lee E. J. (2025). Human endogenous retrovirus-K envelope protein is aberrantly expressed in serous ovarian cancer and promotes chemosensitivity via NF-κB/P-glycoprotein pathway inhibition. J. Ovarian Res. 18. doi: 10.1186/s13048-025-01722-2, PMID: 40616136 PMC12232173

[B50] Paganelli A. Cristofoletti C. Moro F. Corrente A. Colonna L. Scala E. . (2025). Comprehensive overview of cytokine interplay in vitiligo: A decade of meta-analyses systematically reviewed. Life (Basel) 15. doi: 10.3390/life15050684, PMID: 40430113 PMC12112851

[B51] Pasternack N. Doucet-O'Hare T. Johnson K. Paulsen O. Nath A. (2024). Endogenous retroviruses are dysregulated in ALS. iScience 27. doi: 10.1016/j.isci.2024.110147, PMID: 38989463 PMC11233923

[B52] Pathak G. N. Tan I. J. Bai G. Dhillon J. Rao B. K. (2024). Vitiligo: From mechanisms of disease to treatable pathways. Skin Health Dis. 4. doi: 10.1002/ski2.460, PMID: 39624766 PMC11608881

[B53] Perron H. Dougier-Reynaud H.-L. Lomparski C. Lafon M. Liblau R. Lassmann H. . (2013). Human endogenous retrovirus protein activates innate immunity and promotes experimental allergic encephalomyelitis in mice. PLoS One 8, e80128. doi: 10.1371/journal.pone.0080128, PMID: 24324591 PMC3855614

[B54] Perron H. Garson J. A. Bedin F. Beseme F. Paranhos-Baccala G. Komurian-Pradel F. . (1997). Molecular identification of a novel retrovirus repeatedly isolated from patients with multiple sclerosis. The Collaborative Research Group on Multiple Sclerosis. Proc. Natl. Acad. Sci. USA 94, 7583–7588. doi: 10.1073/pnas.94.14.7583, PMID: 9207135 PMC23865

[B55] Perron H. Germi R. Bernard C. Garcia-Montojo M. Deluen C. Farinelli L. . (2012). Human endogenous retrovirus type W envelope expression in blood and brain cells provides new insights into multiple sclerosis disease. Mult. Scler. 18, 1721–1736. doi: 10.1177/1352458512441381, PMID: 22457345 PMC3573672

[B56] Picardo M. Dell'Anna M. L. Ezzedine K. Hamzavi I. Harris J. E. Parsad D. . (2015). Vitiligo. Nat. Rev. Dis. Primers 1. doi: 10.1038/nrdp.2015.11, PMID: 27189851

[B57] Ramasamy R. Joseph B. Whittall T. (2017). Potential molecular mimicry between the human endogenous retrovirus W family envelope proteins and myelin proteins in multiple sclerosis. Immunol. Lett. 183, 79–85. doi: 10.1016/j.imlet.2017.02.003, PMID: 28189601

[B58] Ramirez-Ramirez V. Macias-Islas M. A. Ortiz G. G. Pacheco-Moises F. Torres-Sanchez E. Sorto-Gomez T. . (2013). Efficacy of fish oil on serum of TNF α, IL-1 β, and IL-6 oxidative stress markers in multiple sclerosis treated with interferon beta-1b. Oxid. Med. Cell. Longev. doi: 10.1155/2013/709493, PMID: 23861993 PMC3703725

[B59] Rangel S. C. da Silva M. D. da Silva A. L. dos Santos J. de M. B. Neves L. M. Pedrosa A. . (2022). Human endogenous retroviruses and the inflammatory response: A vicious circle associated with health and illness. Front. Immunol. 13, 1057791. doi: 10.3389/fimmu.2022.1057791, PMID: 36518758 PMC9744114

[B60] Rangel S. C. da Silva M. D. Natrielli Filho D. G. Santos S. N. do Amaral J. B. Victor J. R. . (2024). HERV-W upregulation expression in bipolar disorder and schizophrenia: unraveling potential links to systemic immune/inflammation status. Retrovirology 21, 1–10. doi: 10.1186/s12977-024-00640-3, PMID: 38644495 PMC11034070

[B61] Rasmussen H. B. Geny C. Deforges L. Perron H. Tourdias T. Jauberteau M. O. . (1995). Expression of endogenous retroviruses in blood mononuclear cells and brain tissue from multiple sclerosis patients. Mult. Scler. 1, 82–87. doi: 10.1177/135245859500100205, PMID: 9345457

[B62] Rodrigues L. S. Da Silva Nali L. H. Leal C. O. D. Sabino E. C. Lacerda E. M. Kingdon C. C. . (2019). HERV-K and HERV-W transcriptional activity in myalgic encephalomyelitis/chronic fatigue syndrome. Autoimmun. Highlights 10, 12. doi: 10.1186/s13317-019-0122-8, PMID: 32257068 PMC7065355

[B63] Rolland A. Jouvin-Marche E. Saresella M. Ferrante P. Cavaretta R. Créange A. . (2005). Correlation between disease severity and *in vitro* cytokine production mediated by MSRV (multiple sclerosis associated retroviral element) envelope protein in patients with multiple sclerosis. J. Neuroimmunol. 160, 195–203. doi: 10.1016/j.jneuroim.2004.10.019, PMID: 15710473

[B64] Rolland A. Jouvin-Marche E. Viret C. Faure M. Perron H. Marche P. N. (2006). The envelope protein of a human endogenous retrovirus-W family activates innate immunity through CD14/TLR4 and promotes Th1-like responses. J. Immunol. 176, 7636–7644. doi: 10.4049/jimmunol.176.12.7636, PMID: 16751411

[B65] Sandoval-Cruz M. García-Carrasco M. Sánchez-Porras R. Mendoza-Pinto C. Jiménez-Hernández M. Munguía-Realpozo P. . (2011). Immunopathogenesis of vitiligo. Autoimmun. Rev. 10, 762–765. doi: 10.1016/j.autrev.2011.02.004, PMID: 21334464

[B66] Santos C. A. F. Amirato G. R. Paixão V. Almeida E. B. Do Amaral J. B. Monteiro F. R. . (2023). Association among inflammaging, body composition, physical activity, and physical function tests in physically active women. Front. Med. (Lausanne) 10. doi: 10.3389/fmed.2023.1206989, PMID: 37534321 PMC10390738

[B67] Savino F. Montanari P. Dini M. Pau A. Ferroglio L. Burdisso S. . (2025). Peripheral blood and nasal swabs Type I and III IFNs signature in RSV positive infant with bronchiolitis. J. Immunol. Methods 543. doi: 10.1016/j.jim.2025.113918, PMID: 40759205

[B68] SenGupta D. Tandon R. Vieira R. G. S. Ndhlovu L. C. Lown-Hecht R. Ormsby C. E. . (2011). Strong human endogenous retrovirus-specific T cell responses are associated with control of HIV-1 in chronic infection. J. Virol. 85, 6977–6985. doi: 10.1128/JVI.00179-11, PMID: 21525339 PMC3126607

[B69] Sheth V. M. Guo Y. Qureshi A. A. (2013). Comorbidities associated with vitiligo: a ten-year retrospective study. Dermatology 227, 311–315. doi: 10.1159/000354607, PMID: 24107643

[B70] Silva K. C. N. da Cruz Ballerini A. P. A. Flose B. R. Silva M. D. Pimenta C. A. M. Shio M. T. . (2025). Profile of expression of human endogenous retroviruses and their interplay on inflammatory status in patients with chronic venous disease. Hum. Gene 44, 201411. doi: 10.1016/J.HUMGEN.2025.201411, PMID: 41936479

[B71] Søe K. Andersen T. L. Hobolt-Pedersen A. S. Bjerregaard Bolette B. Larsson L. I. Delaissé J. M. (2011). Involvement of human endogenous retroviral syncytin-1 in human osteoclast fusion. Bone 48, 837–846. doi: 10.1016/j.bone.2010.11.011, PMID: 21111077

[B72] Sotgiu S. Mameli G. Serra C. Zarbo I. Arru G. Dolei A. (2010). Multiple sclerosis-associated retrovirus and progressive disability of multiple sclerosis. Mult. Scler. 16, 1248–1251. doi: 10.1177/1352458510376956, PMID: 20685761

[B73] Speeckaert R. Speeckaert M. De Schepper S. van Geel N. (2017). Biomarkers of disease activity in vitiligo: A systematic review. Autoimmun. Rev. 16, 937–945. doi: 10.1016/j.autrev.2017.07.005, PMID: 28698094

[B74] Sreekumar G. P. Smyth J. R. Ambady S. Ponce de Leon F. A. (2000). Analysis of the effect of endogenous viral genes in the Smyth line chicken model for autoimmune vitiligo. Am. J. Pathol. 156, 1099–1107. doi: 10.1016/S0002-9440(10)64978-4, PMID: 10702426 PMC1876847

[B75] Tamouza R. Meyer U. Lucas A. Richard J. R. Nkam I. Pinot A. . (2023). Patients with psychosis spectrum disorders hospitalized during the COVID-19 pandemic unravel overlooked SARS-CoV-2 past infection clustering with HERV-W ENV expression and chronic inflammation. Transl. Psychiatry 13. doi: 10.1038/s41398-023-02575-3, PMID: 37524719 PMC10390536

[B76] Tang B. Liu Z. Xiong H. Zhang J. Dai J. (2025). IFN-λ: unleashing its potential in disease therapies from acute inflammation regulation to cancer immunotherapy. Immunology 176, 197–214. doi: 10.1111/imm.13954, PMID: 40421666

[B77] Tarlinton R. Wang B. Morandi E. Gran B. Khaiboullin T. Martynova E. . (2020). Differential expression of HERV-W in peripheral blood in multiple sclerosis and healthy patients in two different ethnic groups. Front. Pharmacol. 10, 1645. doi: 10.3389/fphar.2019.01645, PMID: 32076404 PMC7002920

[B78] Ting C. N. Rosenberg M. P. Snow C. M. Samuelson L. C. Meisler M. H. (1992). Endogenous retroviral sequences are required for tissue-specific expression of a human salivary amylase gene. Genes Dev. 6, 1457–1465. doi: 10.1101/gad.6.8.1457, PMID: 1379564

[B79] Tomaszewska K. Kozłowska M. Kaszuba A. Lesiak A. Narbutt J. Zalewska-Janowska A. . (2020). Increased serum levels of IFN-γ, IL-1β, and IL-6 in patients with alopecia areata and nonsegmental vitiligo. Oxid. Med. Cell. Longev. 2020, 5693572. doi: 10.1155/2020/5693572, PMID: 32832001 PMC7421748

[B80] Tovo P. A. Garazzino S. Savino F. Daprà V. Pruccoli G. Dini M. . (2023a). Expressions of type I and III interferons, endogenous retroviruses, TRIM28, and SETDB1 in children with respiratory syncytial virus bronchiolitis. Curr. Issues Mol. Biol. 45, 1197–1217. doi: 10.3390/cimb45020079, PMID: 36826024 PMC9954910

[B81] Tovo P. A. Marozio L. Abbona G. Calvi C. Frezet F. Gambarino S. . (2023b). Pregnancy is associated with impaired transcription of human endogenous retroviruses and of TRIM28 and SETDB1, particularly in mothers affected by multiple sclerosis. Viruses 15. doi: 10.3390/v15030710, PMID: 36992419 PMC10051116

[B82] Upadhya S. Andrade M. J. Shukla V. Rao R. Satyamoorthy K. (2025). Genetic and immune dysregulation in vitiligo: Insights into autoimmune mechanisms and disease pathogenesis. Autoimmun. Rev. 24. doi: 10.1016/j.autrev.2025.103841, PMID: 40466982

[B83] von Elm E. Altman D. G. Egger M. Pocock S. J. Gøtzsche P. C. Vandenbroucke J. P. (2008). The Strengthening the Reporting of Observational Studies in Epidemiology (STROBE) statement: guidelines for reporting observational studies. J. Clin. Epidemiol. 61, 344–349. doi: 10.1016/j.jclinepi.2007.11.008, PMID: 18313558

[B84] Wang D. Gomes M. T. Mo Y. Prohaska C. C. Zhang L. Cheivanambi S. . (2023). Human endogenous retrovirus, SARS-CoV-2, and HIV promote PAH via inflammation and growth stimulation. Int. J. Mol. Sci. 24. doi: 10.3390/ijms24087472, PMID: 37108634 PMC10138839

[B85] Wentzensen N. Coy J. F. Knaebel H.-P. Linnebacher M. Wilz B. Gebert J. . (2007). Expression of an endogenous retroviral sequence from the HERV-H group in gastrointestinal cancers. Int. J. Cancer 121, 1417–1423. doi: 10.1002/ijc.22826, PMID: 17546591

[B86] Zhu M. C. Liu C. G. Wang D. X. Zhan Z. (2015). Detection of serum anti-melanocyte antibodies and identification of related antigens in patients with vitiligo. Genet. Mol. Res. 14, 16060–16073. doi: 10.4238/2015.December.7.19, PMID: 26662399

